# A Meta-Analysis of the Performance of a Blood-Based Exposure Response Gene Signature Across Clinical Studies on the Tobacco Heating System 2.2 (THS 2.2)

**DOI:** 10.3389/fphar.2019.00198

**Published:** 2019-03-26

**Authors:** Florian Martin, Marja Talikka, Nikolai V. Ivanov, Christelle Haziza, Julia Hoeng, Manuel C. Peitsch

**Affiliations:** Philip Morris International Research and Development, Philip Morris Products S.A., Neuchâtel, Switzerland

**Keywords:** gene expression, signature, blood, modified risk tobacco products, smoker

## Abstract

As part of emerging tobacco harm reduction strategies, modified risk tobacco products (MRTP) are being developed to offer alternatives that have the potential to reduce the individual risk and population harm compared with smoking cigarettes for adult smokers who want to continue using tobacco and nicotine products. MRTPs are defined as any tobacco products that are distributed for use to reduce harm or the risk of tobacco-related disease associated with commercially marketed tobacco products. One such candidate MRTP is the Tobacco Heating System (THS) 2.2, which does not burn tobacco but instead heats it, thus producing significantly reduced levels of harmful and potentially harmful constituents compared with cigarettes. The clinical assessment of candidate MRTPs requires the development of exposure-response markers to distinguish current smokers from either nonsmokers or former smokers with high specificity and sensitivity. Toward this end, a whole blood-derived gene signature was previously developed and reported. Four randomized, controlled, open-label, three-arm parallel group reduced exposure clinical studies have been conducted with subjects randomized to three arms: switching from cigarettes to THS 2.2, continuous use of cigarettes, or smoking abstinence. These clinical studies had an investigational period of 5 days in confinement, which was followed by an 85-day ambulatory period in two studies. Here we tested the previously developed blood-derived signature on the samples derived from those clinical studies. We showed that in all four studies, the signature scores were reduced consistently in subjects who either stopped smoking or switched to THS 2.2 compared with subjects who continued smoking cigarettes.

## Introduction

Cigarette smoking is one of the leading causes of preventable human morbidity and mortality, causing serious diseases such as cardiovascular diseases, chronic obstructive pulmonary disease (COPD), and lung cancer. The vast majority of the smoking-related diseases are caused by the toxicants present in cigarette smoke (Farsalinos and Le Houezec, 2015), which are mostly formed during the combustion of tobacco^1^. The United States Surgeon General has stated that the “burden of death and disease from tobacco use in the United States is overwhelmingly caused by cigarettes and other combusted tobacco products” (U.S. Department of Health and Human Services, 2014). Nicotine, while addictive, not risk-free, and an important factor in why people smoke, is not the primary cause of diseases (Tobacco Advisory Group of the Royal College of Physicians, 2000; Benowitz, 2010; U.S. Department of Health and Human Services, 2014).

For decades, the efforts to reduce the harm caused by smoking have been focused on preventing smoking initiation and promoting smoking cessation (Zhu et al., 2012). More recently, tobacco harm reduction (THR) has emerged as a third approach that can help to reduce the adverse effects of smoking. THR is based on switching consumers to less harmful products that emit significantly lower levels of toxicants while providing levels of nicotine comparable to cigarettes (Smith et al., 2016). As noted by McNeil, “Since nicotine itself is not a highly hazardous drug, encouraging smokers to obtain nicotine from sources that do not involve tobacco combustion is a potential means to reduce the morbidity and mortality they sustain, without the need to overcome their addiction to nicotine” (McNeil, 2012). This new approach complements those aimed at reducing smoking prevalence and aims to provide smokers who do not quit with novel tobacco or nicotine-containing products that are substantially less toxic than cigarettes. The United States Family Smoking Prevention and Tobacco Control Act embraces the concept of THR and defines a modified risk tobacco product (MRTP) as any tobacco product that is sold or distributed for use to reduce harm or the risk of tobacco related disease associated with commercially marketed tobacco products (Luke et al., 2011).

MRTPs are designed with the objective to significantly reduce or eliminate the emission of toxicants – so-called harmful and potentially harmful constituents (HPHC) – while preserving the taste, sensory experience, nicotine delivery profile, and ritual characteristics of cigarettes as much as possible. The aim of these products is to both reduce the risk of smoking-related disease and allow current adult smokers to switch to them. While still containing tobacco, novel heated tobacco products generally emit significantly lower levels of HPHCs than cigarettes (Tobacco Advisory Group of the Royal College of Physicians, 2016; Farsalinos et al., 2017). One such candidate MRTP is the Tobacco Heating System (THS) 2.2 developed by Philip Morris International, which has been launched in several European and Asian markets. Other similar products exist and are becoming an important part of current harm reduction strategies (Tobacco Advisory Group of the Royal College of Physicians, 2016; Murphy et al., 2017).

Tobacco Heating System 2.2 is a *heat-not-burn* product that heats tobacco at a temperature below that required to initiate combustion (Smith et al., 2016). THS 2.2 consists of three components: (1) a tobacco stick, a novel patent-pending tobacco product; (2) a holder, into which the tobacco stick is inserted, which heats the tobacco by means of an electronically controlled heating blade, and (3) a charger, which is used to recharge the holder after each use. To operate the THS 2.2, the user inserts a tobacco stick into the holder and turns on the device by means of a switch. This initiates the heating of the tobacco via the heating blade inserted into the tobacco plug. Heat is supplied to the tobacco stick for a fixed period of approximately 6 min and allows up to 14 puffs to be taken during that time. The temperature of the heating blade is controlled carefully, and the energy supply to the blade is cut if its operating temperature exceeds 350°C (Smith et al., 2016).

Tobacco Heating System 2.2, which was designed to heat rather than burn tobacco, emits > 90% lower levels of HPHCs than cigarettes (Forster et al., 2015; Schaller et al., 2016b; Bekki et al., 2017). As a consequence, smokers who switch completely to THS 2.2 use should be exposed to significantly lower levels of HPHCs.

To test this hypothesis, four randomized, controlled, open-label, three-arm parallel group clinical studies have been conducted to determine whether switching from smoking to THS 2.2 use reduces exposure to HPHCs and to compare the reductions in exposure with those observed when subjects quit for the duration of the studies. The exposure to HPHCs was quantified using Biomarkers of Exposure (BoExp) to specific smoke constituents, and the four studies demonstrated consistently that study participants who switched from cigarette smoking to THS 2.2 used were exposed to significantly lower levels of HPHCs than those who continued to smoke cigarettes. Furthermore, these studies showed that the effects of switching were similar to those caused by smoking abstinence (SA) (Haziza et al., 2016a,b; Ludicke et al., 2018a,b; Haziza et al., 2019).

While BoExp are valuable tools to quantify the body’s exposure to specific HPHCs, they do not capture the biological responses elicited by the exposure to HPHCs. Systems toxicology aims to achieve a global understanding of the exposure response and utilizes high-throughput technologies for extensive molecular measurements (Sturla et al., 2014; Hartung et al., 2017). In the context of smoking, gene expression profiling has revealed mechanistic insights into the disease-related changes that occur in the respiratory track of smokers (Spira et al., 2004, 2007). Gene expression profiling can also be used to develop signatures that are characteristic to certain subject groups and have predictive and prognostic value. A recent analysis of existing gene signatures for primary lung adenocarcinoma showed a significant classification agreement between the signatures (Ringnér and Staaf, 2016).

Even though it is not the primary tissue affected by smoking, whole blood is an attractive matrix for gene expression profiling. Blood is easy to acquire and reflects the smoking-induced changes in the systemic immune response (Jensen et al., 1998; Wang et al., 2013; Hoonhorst et al., 2014). The challenge is that the molecular changes are difficult to turn into mechanistic insights because of the heterogeneity of the cellular composition of whole blood. Therefore, the value of blood-based gene expression profiling resides in the genes, in which expression pattern is distinctively representative of an exposure or a disease. For example, blood-based gene signatures have been shown to reflect different lung pathologies in patients (Showe et al., 2009; Rotunno et al., 2011; Zander et al., 2011; Bloom et al., 2013; DePianto et al., 2014; Huang et al., 2015) and even toxicant exposures (LaBreche et al., 2011; Beineke et al., 2012; Joseph et al., 2013; Bushel et al., 2016).

We have recently developed a blood-based gene signature that can distinguish current smokers (CS) from either nonsmokers (NS) or former smokers (FS) with high specificity and sensitivity (Martin et al., 2015). This smoke exposure response signature (SERS) of 11 genes (*LRRN3, SASH1, PALLD, RGL1, TNFRSF17, CDKN1C, IGJ, RRM2, ID3, SERPING1*, and *FUCA1*) has since been verified in the systems biology verification Industrial Methodology for PROcess VErification in Research (sbv IMPROVER) Systems Toxicology Computational Challenge. The genes proposed by the best performers overlapped remarkably with our gene signature (Belcastro et al., 2017; Poussin et al., 2017). The SERS could distinguish CS from NS with remarkable accuracy (Martin et al., 2015). Furthermore, the prediction scores from the training and validation cohort showed a clear separation between the FS (NS, respectively) smokers and the CS, allowing for a quantitative monitoring of smoking exposure. In these studies, no separation between FS (at least one year after quitting smoking) and NS was observed (Belcastro et al., 2017; Poussin et al., 2017).

To test this quantitative monitoring of smoking exposure, the SERS was further applied to blood samples collected from one of our 5-day reduced exposure clinical studies (NCT01959932, ZRHR-REX-C-03-EU) (Haziza et al., 2016b; Martin et al., 2016). The results obtained for the 5-day cessation cohort samples confirmed that the signature scores could serve as a quantitative biomarker. Once applied to the samples from the associated 5-day THS 2.2 switching arm, the reduction in score was comparable (Martin et al., 2016). The signature performed remarkably well in predicting significant reductions in exposure response within just 5 days after subjects switched to THS 2.2 or abstained from smoking. In this study, continued smoking served as a negative control (no change expected), while smoking abstinence, which removes the exposure completely, served as a positive control.

Here we have included the three other clinical reduced exposure studies conducted with THS 2.2 (NCT01970982, NCT01970995, and NCT01989156) to test the performance of the SERS in smokers who switched to THS 2.2 or abstained. One of the studies was similar to that reported in Haziza et al. (2016b) with exposure for 5 days in confinement, and two others included an additional 85-day ambulatory period. The detailed presentation of the primary and secondary endpoints of the studies is available in the study publications (Haziza et al., 2016a,b, 2019; Ludicke et al., 2018a,b).

## Materials and Methods

### Reduced Exposure Studies

#### *Ad libitum* Product Use for Five Days (ZRHR-REXC-03-EU and ZRHR-REXC-04-JP)

**FIGURE 1:**
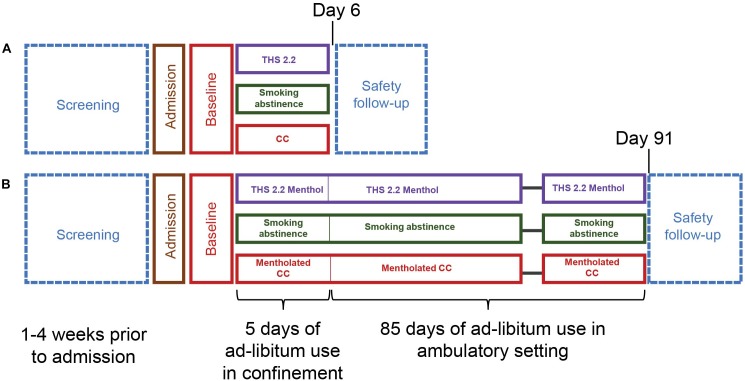
Reduced exposure study designs. **(A)** Study design for the confinement studies ZRHR-REXC-04-JP and ZRHR-REXC-03-EU. **(B)** Study design for the ambulatory studies ZRHM-REXA-07-JP and ZRHM-REXA-08-US.

These randomized, open-label, parallel group reduced exposure studies included THS 2.2 nonmenthol (*ad libitum* use), cigarette (*ad libitum* use), and SA arms with a 5-day investigational exposure period that was subsequent to a 2-day baseline period during which all subjects smoked their own brand of cigarettes. In these studies, continued smoking was considered as the negative control arm (“no-effect”) while the SA arm, for which smoke exposure was removed, as the positive control (”maximum effect”). After discharge, or in case of an early discontinuation, participants entered a 7-day safety follow-up period for recording of spontaneously reported new adverse events (AE), serious adverse events (SAE), or follow up of any ongoing AEs/SAEs that could have occurred during the study (Figure 1A).

The aim of these studies was to assess the reduction in the levels of BoExp to selected HPHCs following switching to THS 2.2 in an optimal, clinical setting where compliance to arm allocation was controlled by the site staff. Exposure to nicotine and subjective effects related to smoking (urge to smoke, withdrawal symptoms, and product evaluation) were assessed, and safety was monitored. These studies, conducted in Europe (ZRHR-REXC-03-EU) and Japan (ZRHR-REXC-04-JP), were registered on clinicaltrials.gov with the numbers NCT01959932 and NCT01970982, respectively. These studies demonstrated that the exposure to 15 HPHCs (Table 1) was significantly reduced upon switching from cigarette smoking to THS 2.2 use, and that these reductions were of a similar magnitude than those observed in study participants who abstained from smoking for the duration of the study (Haziza et al., 2016a,b). No serious adverse events related to THS 2.2 arms were reported and overall, the incidence of adverse events was comparable in the THS 2.2 and CC arms.

**Table 1:** HPHC yields and associated biomarkers of exposure reported in four clinical studies.

HPHC [Organ class toxicity^1^]		3R4F^2^	THS2.2 FR1^2^	THS2.2 FR1 M^2^	Biomarker of Exposure [Matrix]
1	1,3-butadiene [CA, RT, RDT]	63.8 ± 3.5 μg/stick	0.294 ± 0.042 μg/stick	0.265 ± 0.024 μg/stick	Monohydroxybutenylmercapturic acid (MHBMA) [Urine]
2	1-aminonaphthalene [CA]	20.8 ± 1.3 ng/stick	0.077 ng/stick	0.086 ng/stick	1-Aminonaphthalene (1-NA) [Urine]
3	2-aminonaphthalene [CA]	11.0 ± 0.6 ng/stick	0.046 ± 0.008 ng/stick	<0.035 ng/stick	2-Aminonaphthalene (2-NA) [Urine]
4	4-(methylnitrosamino)-1-(3-pyridyl)-1-butanone (NNK) [CA]	266 ± 15 ng/stick	6.7 ± 0.6 ng/stick	5.9 ± 0.4 ng/stick	Total 4-(methylnitrosamino)- 1-(3-pyridyl)-1-butanol (total NNAL) [Urine]
5	4-aminobiphenyl [CA]	3.26 ± 0.12 ng/stick	<0.051 ng/stick	<0.051 ng/stick	4-Aminobiphenyl (4-ABP) [Urine]
6	Acrolein [RT, CT]	154 ± 20 μg/stick	11.30 ± 2.36 μg/stick	9.15 ± 0.43 μg/stick	3-Hydroxypropyl- mercapturic acid (3-HPMA) [Urine]
7	Acrylonitrile [CA, RT]	31.9 ± 1.8 μg/stick	0.258 ± 0.041 μg/stick	0.220 ± 0.014 μg/stick	2-Cyanoethyl Mercapturic acid (CEMA) [Urine]
8	Benzene [CA, CT, RDT]	97.6 ± 4.7 μg/stick	0.649 ± 0.074 μg/stick	0.640 ± 0.040 μg/stick	S-Phenyl-mercapturic acid (S-PMA) [Urine]
9	Benzo[a]pyrene [CA]	14.2 ± 0.3 ng/stick	<1.00 ng/stick	1.29 ± 0.10 ng/stick	Total 3-Hydroxy Benzopyrene (3-OH-B[a]P)[Urine]
10	Carbon monoxide [RDT, CT]	32.8 ± 2.4 mg/stick	0.531 ± 0.068 mg/stick	0.594 ± 0.110 mg/stick	Carboxyhemoglobin (COHb) [Blood]
11	Crotonaldehyde [CA]	68.8 ± 14.4 μg/stick	4.14 ± 0.23 μg/stick	3.24 ± 0.21 μg/stick	3-Hydroxy-1-methyl propyl-mercapturic acid (3-HMPMA) [Urine]
12	Ethylene oxide [CA, RT, RDT]	29.4 ± 2.0 μg/stick	0.201 ± 0.014 μg/stick	0.202 ± 0.013 μg/stick	2-Hydroxyethyl-mercapturic acid (HEMA) [Urine]
13	N-nitrosonornicotine (NNN) [CA]	309 ± 41 ng/stick	17.2 ± 1.25 ng/stick	13.7 ± 1.21 ng/stick	Total N-nitrosonornicotine (total NNN) [Urine]
14	*o*-toluidine [CA]	85.5 ± 2.7 ng/stick	1.260 ± 0.18 ng/stick	0.777 ± 0.287 ng/stick	o-Toluidine (o-tol) [Urine]
15	Pyrene [Indicator of CA substances]	87.3 ± 2.5 ng/stick	<5.00 ng/stick	9.06 ± 0.68 ng/stick	Total 1-hydroxypyrene (1-OHP) [Urine]


*All markers were measured in 24h urine, with the exception of carboxyhemoglobin which was measured in blood samples. Total 4-(methylnitrosamino)-1-(3-pyridyl)-1-butanol was determined as the molar sum of 4-(methylnitrosamino)-1-(3-pyridy1)-1-butanol and its O-glucuronide conjugate and Total 1-hydroxypyrene was determined as the molar sum of 1-hydroxypyrene and its glucuronide and sulfate conjugates. HPHC yields from 3R4F and THS 2.2 FR1 were obtained under HCI machine-smoking conditions and expressed on a per-cigarette/tobacco stick basis.*

*3R4F = Reference combustible cigarette from University of Kentucky, AD = addictive, CA = carcinogen, CT = cardiovascular toxicant, RT = respiratory toxicant, RDT = reproductive and developmental toxicant, THS2.2 FR1 = THS 2.2 heatsticks, THS2.2 FR1 M = mentholated version of THS2.2 FR1.*

*^*1*^Organ class toxicities from FDA list of HPHCs (US Food and Drug Administration, 2012).*

*^*2*^Analyte yields from THS2.2 FR1, THS2.2 FR1 M, and 3R4F obtained under HCI machine-smoking conditions and expressed on a per-cigarette/tobacco stick basis published in Schaller et al. (2016a).*

#### *Ad libitum* Product Use for Five Days in Confinement Followed by 85 Days in Ambulatory Setting (ZRHM-REXA-07-JP, ZRHM-REXA-08-US)

These randomized, open-labels, parallel group reduced exposure studies included THS 2.2 in its mentholated version (*ad libitum* use), cigarette (*ad libitum* use), and SA arms and had two distinct periods: a 5-day confinement investigational exposure period to the investigational product in confinement followed by an 85-day ambulatory investigational exposure period (Figure 1B). In these studies, continued smoking was considered as the negative control arm (“no-effect”) while the SA arm, for which smoke exposure was removed, as the positive control (“maximum effect”).

The aim of these studies was also to assess the reduction in the levels of BoExp to HPHCs following switching to THS 2.2. The same set of BoExp as in the confinement studies described above were measured (Table 1). The additional ambulatory study period was designed to assess if the reductions in exposure observed in a confined setting were sustained in an ambulatory period, a more “real-life” setting where confounding factors, such as environment, diet, passive smoking, and use of cigarettes in combination with THS 2.2 (dual-use), could influence the levels of exposure to HPHCs. Furthermore, these studies provided continued insights in the understanding of product use and acceptance and the safety associated with product use over a prolonged period. Product use was monitored in a log (confinement period with strict product dispensation) and was monitored during the ambulatory period by self-reporting of product use in an electronic diary. These studies were conducted in Japan (ZRHM-REXA-07-JP) and the U.S. (ZRHM-REXA-08-US) and registered on clinicaltrials.gov with the numbers NCT01970995 and NCT01989156, respectively. These studies demonstrated that the exposure to 15 HPHCs (Table 1) was significantly reduced upon switching from mentholated cigarette smoking to mentholated THS 2.2 use after 5 days of use in confinement (Ludicke et al., 2018a; Haziza et al., 2019). Furthermore, these studies showed that these reductions were sustained during the ambulatory period after 3 months in smokers who switched from cigarette smoking to THS 2.2 use (Ludicke et al., 2018b; Haziza et al., 2019). In addition, changes in clinical risk endpoints followed the direction of smoking cessation (Ludicke et al., 2018b). No serious adverse events related to THS 2.2 arms were reported and overall, the incidence of adverse events was comparable in the THS 2.2 and CC arms.

All studies were conducted according to the principles of Good Clinical Practices, and an ethics committee approved each study. All participants provided written informed consent before participation in the studies. An additional informed consent for blood sample collection for transcriptomics profiling was provided to each participant. Participants that did not provide this specific informed consent could still participate in the main study. The details of the inclusion and exclusion criteria of the subjects are provided under the clinical protocol for each study at clinicaltrials.gov.

### Sample Collection and Handling

Whole blood was collected for transcriptomics profiling from subjects who provided the additional informed consent. For the four clinical studies, samples were collected at baseline and after 5 days. Additional samples were collected after 3 months for the two ambulatory studies. The sample collection was done in PAXgene RNA blood tubes (preanalytix: Cat: 762165 BD) according to the reagent manufacturer’s instructions. The samples were frozen and shipped to bio-banking. All data related to these blood samples were anonymized. Anonymized data and samples were initially single or double coded, and the association between the unique code(s) and the subjects’ identifiers was deleted. In the studies ZRHR-REXC-03-EU, ZRHR-REXC-04-JP, ZRHM-REXA-07-JP samples from all time points were collected for each subject. In the study ZRHM-REXA-08-US, samples from the latest time point was missing for only 6 subjects, 2 in the SA arm and 4 in the THS 2.2 arm.

### RNA Isolation

Before RNA extraction, the samples underwent a complete block randomization, where the blocking factor was defined by the unique subject ID to group samples collected for a single subject together. The PAXgene Blood miRNA Kit (Qiagen) was used to isolate the total RNAs from the blood samples according to the instructions provided by the manufacturer. The absorbance at 230, 260, and 280 nm (NanoDrop ND1000; Thermo Fisher Scientific, Waltham, MA, United States) was measured to evaluate the concentration and purity of RNA samples. The Agilent 2100 Bioanalyzer (Agilent Technologies, Santa Clara, CA, United States) was used to determine RNA integrity. Samples with an RNA integrity number greater than six were used for further analysis.

### RNA Preparation and Affymetrix Hybridization and Data Processing

Eighty nanograms of RNA and the Ovation^®^ Whole Blood Reagent and Ovation^®^ RNA Amplification System V2 (NuGEN, San Carlos, CA, United States) were used to prepare Affymetrix probe sets targeting the 3’transcript ends. The SpectraMax UV reader (Molecular Devices, Santa Clara, CA, United States) was used to quantify the amplified cDNA, and the quality was assessed by the size of unfragmented cDNA using a Fragment Analyzer (Ankeny, IA, United States). The size distribution of the final fragmented and biotinylated product was monitored using an Agilent 2100 Bioanalyzer (Agilent Technologies). Hybridization of the labeled cDNA fragments to the GeneChip^®^ Human Genome U133 Plus 2.0 Array (Affymetrix) was done according to the instructions provided by the manufacturer.

### Affymetrix Data Analysis

Using the ReadAffy function of the *affy* package (Gautier et al., 2004) from the Bioconductor suite of microarray analysis tools (Gentleman et al., 2004) available for the R statistical environment (R Development Core Team, 2007), the raw data file (.CEL) were read and analyzed using the custom Chip Description File environment HGU133Plus2_Hs_ENTREZG v16 in conjunction to a frozen-Robust Microarray Analysis (McCall et al., 2010) normalization, for all arrays passing quality control checks (base on the normalized-unscaled standard error (NUSE) median, pseudo-images and the median absolute relative log expression (MARLE) (Bolstad et al., 2003; Gautier et al., 2004). A longitudinal linear model was fitted to quantify the evolution of gene expression over time for each gene. The associated *p*-values were based on moderated *t*-statistics (Smyth, 2005) and adjusted by the Benjamini-Hochberg false discovery rate method for correcting multiple testing effects.

Data are available from ArrayExpress^2^ for the following accession number: E-MTAB-5332 (ZRHR-REXC-04-JP), E-MTAB-5333 (ZRHR-REXC-03-EU), E-MTAB-6559 (ZRHM-REXA-07-JP) and E-MTAB-6558 (ZRHM-REXA-08-US).

### Individual Sample Prediction

The signature and its prediction model, described in our previous publication (Martin et al., 2015), were applied to the generated gene expression data. Two datasets, one from whole blood samples (GSE15289) and the other from peripheral blood mononuclear cells (PBMC) (GSE42057), were leveraged for deriving the signature. The data of those two studies were obtained from the National Center for Biotechnology Information Gene Expression Omnibus^3^ and processed in the same way as described above (Martin et al., 2015). Whole blood samples of the NOWAC study (GSE15289) (Dumeaux et al., 2010) were obtained from post-menopausal women aged between 48 and 63 years, including 211 NS and 74 CS. The PBMC samples collected from Bahr et al. study (GSE42057) (Bahr et al., 2013) were obtained from non-Hispanic Caucasian subjects, either CS or FS, for a total of 42 control subjects and 94 subjects with COPD of varying severity. The signature and its linear prediction model were then derived using the E-MTAB-5279 dataset by conducting the gene selection by the genes exhibiting strong changes in the average expression between CS and NS or FS samples in both datasets, GSE15289 and GSE42057 (Martin et al., 2015). Finally, the signature was validated using the gene expression from the study E-MTAB-5278 (Martin et al., 2015).

In addition to the individual predictions from the model, a relative decrease of the signature score in the three arms (paired by patient ID) was computed to compare the signature response relative to the decrease observed between prediction of CS samples and FS samples in the training dataset, E-MTAB-5279. A value of 100% is achieved when the decrease in signature score is equivalent to the difference between the 90th quantile of the CS and the 10th quantile of the FS in the E-MTAB-5279 cohort, while 0% indicates a prediction score similar to a score from the 90th quantile of the CS arm. This quantifies the reduction in signature response achieved in the different arms. The relative decrease in signature score was the primary endpoint of this study. Additionally, the associated primary comparisons were the relative decreases in the SA and THS 2.2 arms and were all expected to show a significant decrease. In this context, following the intersection-union test (IUT) method (Berger, 1997), the global null hypothesis is rejected at a 0.05 type I error if and only if *all* the individual null hypotheses are rejected at a 0.05 type I error. This implies a controlled overall type I error rate without the need for a multiplicity adjustment.

### Meta-Analysis

The relative decreases from the four studies were analyzed further through meta-analysis by fitting random effect models using the inverse-variance method. Residual heterogeneities were estimated by restricted maximum likelihood (REML) (Viechtbauer, 2005; Konstantopoulos and Hedges, 2009) and Wald-type confidence intervals were derived. The analysis was stratified by study arm and period (confinement and ambulatory respectively). The analysis was performed in *R* using the *metafor* package (Viechtbauer, 2005). Meta-analysis results are displayed as a forest plot.

## Results

**FIGURE 2:**
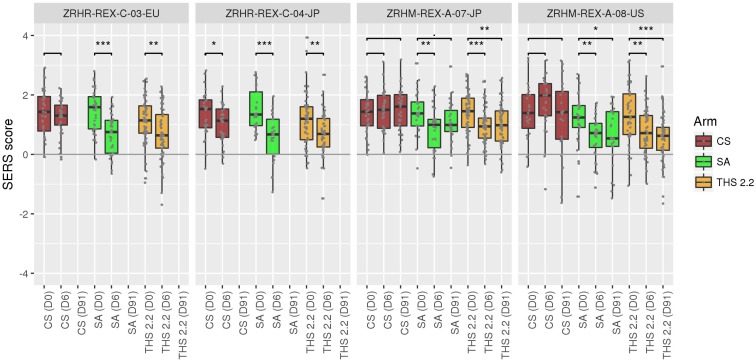
Predictions scores for each sample in the four studies at each time point. Significant decrease (*t*-test *p*-value < 0.05) is observed between Day 6 and baseline for all the SA and THS arms, with the exception of ZRHM-REX-A-07-JP Day 91–Day 0, for which the *p*-value is < 0.1. Comparisons are exploratory tests and were not adjusted for multiple testing. CS: Current smokers; FS: Former smokers; NS: Never smokers [as defined in Martin et al. (2015)]; SA: Smoking abstinence; THS: Tobacco Heating System 2.2 use. D0: Day 0 (baseline); D6: Day 6; D91: Day 91. “.” indicate a *t*-test *p*-value below 0.1, ^∗^: below 0.05, ^∗∗^: below 0.01, ^∗∗∗^: below 0.001. *T*-test are two-sample unpaired, unequal variance *t*-tests (Welch’s *t*-test).

We derived prediction scores for all RNA samples from every study, arm, subject, and sampling time point (Figure 2) for which the array quality control criteria were met (ZRHR-REXC-03-EU: 268 out of 304, ZRHR-REXC-04-JP: 228 out of 302, ZRHM-REXA-07-JP: 378 out of 402, and ZRHM-REXA-08-US: 296 out of 320). For each sampling time point and each subject enrolled, the difference in score to baseline (Day 0) was computed (see Materials and Methods). This difference quantifies the reduction in score of the prediction model, moving away from the smoking status (CS). It was then normalized to the expected difference observed in the training dataset E-MTAB-5279 between a CS sample and an FS sample. The FS used in this training cohort had quit smoking for at least 2 years and had smoked at least 10 cigarettes daily for at least 3 years. To avoid this normalizing range to be too sensitive, the 90th quantile of the CS sample scores and 10th quantile of the FS scores were used. If no change is observed between Day 6 (Day 91, respectively) and baseline (Day 0), it will be close to zero, while it will approach –100% if the score at Day 6 (Day 91, respectively) is comparable to the (almost) maximal change observed between CS and FS.

**FIGURE 3:**
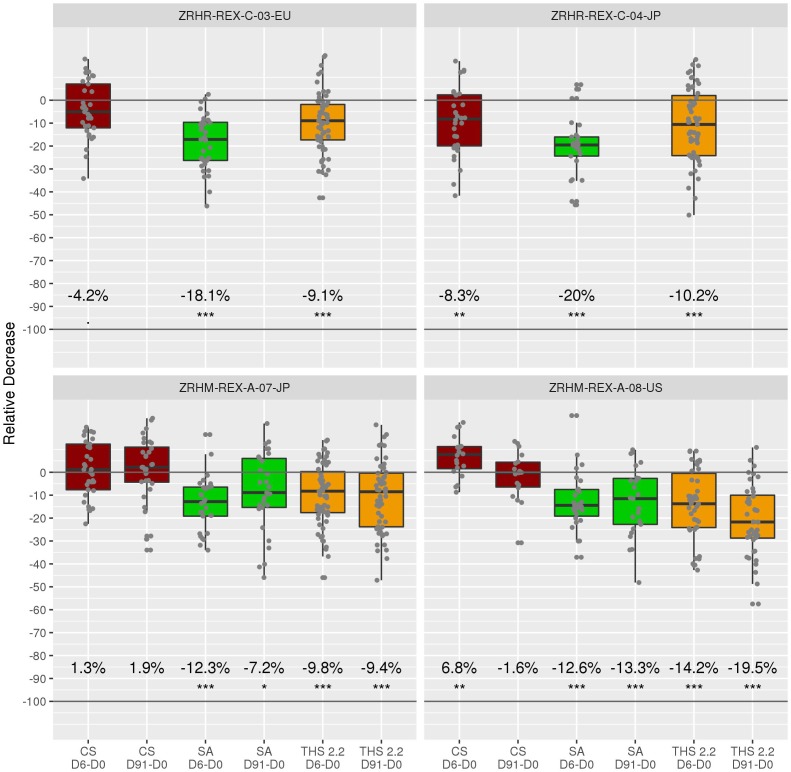
Relative reduction for each subject across arms and studies. The primary comparisons of interest are the relative decreases in the SA and THS arms and are all expected to show a decrease. In this context, following the intersection-union test method, an overall type I error rate of 0.05 is guaranteed without the need for a multiplicity adjustment. The average decrease is indicated under the boxes. “.” indicate a *t*-test *p*-value below 0.1, ^∗^: below 0.05, ^∗∗^: below 0.01, ^∗∗∗^: below 0.001. D0: Day 0 (baseline); D6: Day 6; D91: Day 91. *T*-test refers to a one sample *t*-test on the baseline adjusted values (per subject).

For the confinement periods (up to Day 6), the average decreases for quitting were 18.1, 20, 12.3, and 12.6% for the ZRHR-REXC-03-EU, ZRHR-REXC-04-JP, ZRHM-REXA-07-JP, and ZRHM-REXA-08-US studies, respectively. For the longer period, 7.2 and 13.3% reductions were observed in ZRHM-REXA-07-JP and ZRHM-REXA-08-US, respectively (Figure 3).

The switching arm, in which subjects were asked to use THS 2.2 (or THS2.2M), exhibited decreases of 9.1, 10.2, 9.8, and 14.2% for the confinement period, while 9.4 and 19.5% reductions were observed when followed by the ambulatory period.

Over the different periods and studies, no significant changes were observed for the CS arm, as expected.

**FIGURE 4:**
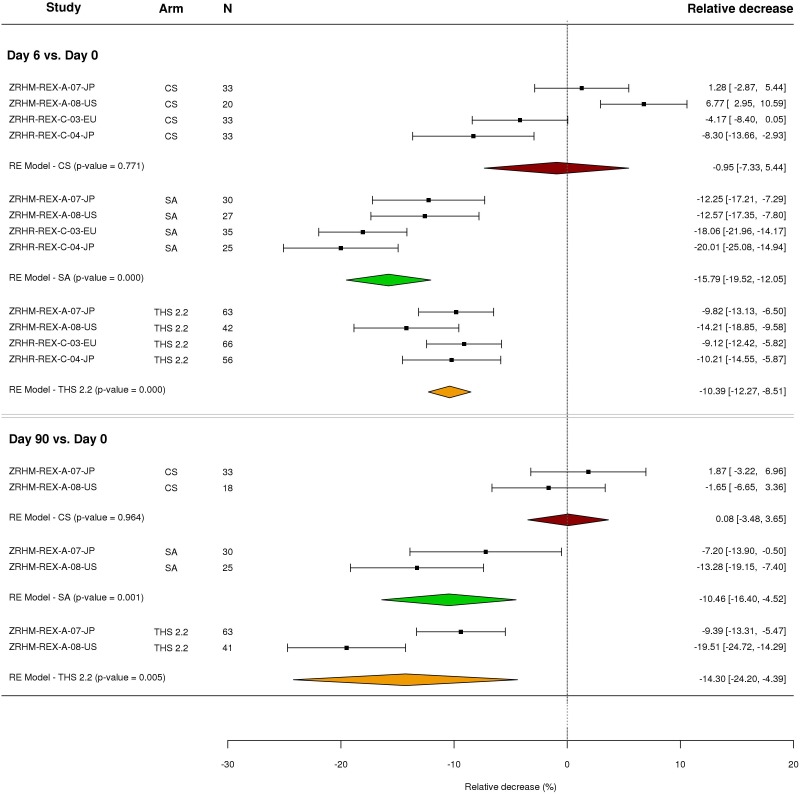
Forest plot of the relative decreases in signature scores for the three arms and two time points across the four studies. Meta-analysis of the relative decrease of the signature score in the three arms (paired by subject) and two periods were performed with a random effect (RE) model (see Materials and Methods). The number (N) of paired differences in each arm and period are shown in the third column. For each study, arm and period, relative decreases and their confidence intervals (normal approximation) are shown on the right-hand side of the plot.

To summarize the observed changes, meta-analyses of the relative decrease were performed for each period and arm (Figure 4). The change in the SA arm is more important than in the THS 2.2 arm for the confinement period, while the opposite trend was observed in the longer period [confinement followed by ambulatory (see discussion)]. All the results indicate a significant decrease for both the THS 2.2 (*p*-value < 1e-4 for the confinement period, 0.004 for the longer period) and SA arms (*p*-value < 1e-4 for both periods) as opposed to the CS arm, which was not expected to exhibit any difference.

## Discussion

Tobacco Heating System 2.2 is a heated tobacco product that emits > 90% lower levels of HPHCs than cigarettes (Schaller et al., 2016b; Tobacco Advisory Group of the Royal College of Physicians, 2016; Farsalinos et al., 2017). This reduction in emission translates into a reduction in exposure among clinical study participants who switched to THS 2.2 compared with those who continued to smoke cigarettes in four clinical reduced exposure studies (Haziza et al., 2016a,b; Ludicke et al., 2018a,b). These reductions were similar to those caused by SA.

Here, we report on a meta-analysis of the performance of the blood-based SERS in the four clinical studies conducted to evaluate the HPHC exposure reduction in smokers who switched from cigarettes to THS 2.2. In a previous article, we showed that after only 5 days, the SERS was able to distinguish between study participants who switched to THS 2.2 and those who continued to smoke cigarettes. During this short period, while the SERS separated the THS 2.2 arm from the continuous cigarette smoking arm, it did not reach the low linear discriminant analysis (LDA) scores observed following long-term smoking cessation (Martin et al., 2015).

Surprisingly, the LDA values for the THS 2.2 users tended to be closer to the FS’ LDA values after the ambulatory period, compared with those who were in the SA arm, in studies ZRHM-REX-A-07 and ZRHM-REX-A-08. This was largely driven by the higher variability observed in the scores for the SA arm over the ambulatory period. The higher LDA score in the SA arms was likely due to non-compliance (i.e., cigarette use) during the ambulatory period.

Blood-based gene signatures, such as the one presented here, could provide a means to test compliance in clinical studies to obtain a reliable measure of reduced exposure during switching to MRTPs or SA. As Affymetrix microarray profiling is rather cost- and labor-intensive and not the most sensitive of available gene expression technologies, quantitative reverse transcription polymerase chain reaction could provide an alternative testing method that has already been tested and described for the gene signature in Martin et al. (2015). The blood-based gene signature could also be transferred to an easy-to-use and economic kit for smaller blood volumes to facilitate clinical use. Such kits and technologies already exist to evaluate radiation exposure (Lucas et al., 2014; Kim et al., 2015). These kits are simple to use at different medical sites and even by subjects in their own homes. Our blood signature is suitable for the development of a smoking status assay leveraging technology, such as the DxDirect^®^, that enables low-cost, high-throughput testing from a few drops of stabilized blood, entirely omitting the RNA isolation step.

While the SERS can serve as a quantitative biomarker, the linearity with respect to the time from quitting smoking and the response score may not be guaranteed. Indeed, the reduction in scores for the SA arm after 91 days compared with the scores at Day 6 is roughly similar. This may be due to a lack of linearity, differences in the confinement and ambulatory settings, or a combination of both.

Nevertheless, gene expression-based signatures, as opposed to detecting a single metabolite from a given HPHC, are linked to the systemic response to exposure and could be able to capture longer term effects. The dynamic ranges observed from the training (E-MTAB-5279) (quitting time of at least 2 years) and validation cohorts (E-MTAB-5278) indicate that the half-life of the SERS biomarker is likely to be more than 3 months. This desirable feature for characterizing smoking exposure response reduction may reflect a slow change in the composition of the blood cell population between CS and NS. For example, LRRN3 is also expressed in T lymphocytes, and its high expression was shown to correlate with a less-senescent T cell phenotype (Chou et al., 2013).

To further unravel the functional relationship between quitting time and SERS scores, samples from an ongoing clinical study (registered in clinicaltrials.gov with the number NCT02432729), comprising a one-year quitting arm, will be leveraged.

## Conclusion

To conclude, the small signature consisting of only 11 genes was applied on the blood transcriptome of subjects enrolled in four clinical studies. The resulting scores showed a consistent reduced exposure response in subjects who either stopped smoking or switched to THS 2.2 (including the mentholated version), compared with subjects who continued smoking cigarettes. With its remarkable capacity to indicate the smoking status of an individual, the signature could be further employed in the development of a low-cost and easy-to-use testing kit for small blood volumes.

## Author Contributions

MP and JH conceived the study. FM processed and analyzed the data and wrote the first draft of the manuscript. MT and CH wrote sections of the manuscript. NI managed the laboratory producing of the data. JH contributed to the writing of the manuscript and provided the resources. MP wrote the manuscript and supervised the work. All authors contributed to manuscript revision, read and approved the submitted version.

## Conflict of Interest Statement

All authors are employees of Philip Morris International.

## Footnotes

1 While most toxicants are formed by combustion processes, tobacco-specific nitrosamines are released from the tobacco.

2 https://www.ebi.ac.uk/arrayexpress/

3 http://www.ncbi.nlm.nih.gov/gds/?term=GEO

## Abbreviations

AE1: adverse event
BoExp: biomarker of exposure
COPD: chronic obstructive pulmonary disease
CS: current smoker
FS: former smoker
HPHC: harmful and potentially harmful constituent
LDA: linear discriminant analysis
MRTP: modified risk tobacco product
NS: nonsmoker
PBMC: peripheral blood mononuclear cells
SA: smoking abstinence
SAE: serious adverse event
ERS: smoking exposure response signature
THR: tobacco harm reduction
THS 2.2: Tobacco Heating System 2.2

## References

[B1] Bahr T. M. Hughes G. J. Armstrong M. Reisdorph R. Coldren C. D. Edwards M. G. (2013). Peripheral blood mononuclear cell gene expression in chronic obstructive pulmonary disease. *Am. J. Respir. Cell Mol. Biol.* 49 316–323. 10.1165/rcmb.2012-0230OC 23590301 PMC3824029

[B2] Beineke P. Fitch K. Tao H. Elashoff M. R. Rosenberg S. Kraus W. E. (2012). A whole blood gene expression-based signature for smoking status. *BMC Med. Genomics* 5:58. 10.1186/1755-8794-5-58 23210427 PMC3538056

[B3] Bekki K. Inaba Y. Uchiyama S. Kunugita N. (2017). Comparison of chemicals in mainstream smoke in heat-not-burn tobacco and combustion cigarettes. *J. UOEH* 39 201–207. 10.7888/juoeh.39.201 28904270

[B4] Belcastro V. Poussin C. Xiang Y. Giordano M. Tripathi K. P. Boda A. (2017). The sbv IMPROVER systems toxicology computational challenge: identification of human and species-independent blood response markers as predictors of smoking exposure and cessation status. *Comput. Toxicol.* 5 38–51. 10.1016/j.comtox.2017.07.004 30221212 PMC6136260

[B5] Benowitz N. L. (2010). Nicotine addiction. *New Engl. J. Med.* 362 2295–2303. 10.1056/NEJMra0809890 20554984 PMC2928221

[B6] Berger R. L. (1997). “Likelihood ratio tests and intersection-union tests,” in *Advances in Statistical Decision Theory and Applications*, eds Panchapakesan S. Balakrishnan N. (Cambridge, MA: Birkhauser Boston Inc.), 225–237. 10.1007/978-1-4612-2308-5_15

[B7] Bloom C. I. Graham C. M. Berry M. P. Rozakeas F. Redford P. S. Wang Y. (2013). Transcriptional blood signatures distinguish pulmonary tuberculosis, pulmonary sarcoidosis, pneumonias and lung cancers. *PLoS One* 8:e70630. 10.1371/journal.pone.0070630 23940611 PMC3734176

[B8] Bolstad B. M. Irizarry R. A. Åstrand M. Speed T. P. (2003). A comparison of normalization methods for high density oligonucleotide array data based on variance and bias. *Bioinformatics* 19 185–193. 10.1093/bioinformatics/19.2.185 12538238

[B9] Bushel P. Fannin R. Gerrish K. Watkins P. Paules R. (2016). Blood gene expression profiling of an early acetaminophen response. *Pharmacogenomics J.* 17 230–236. 10.1038/tpj.2016.8 26927286 PMC5782799

[B10] Chou J. P. Ramirez C. M. Wu J. E. Effros R. B. (2013). Accelerated aging in HIV/AIDS: novel biomarkers of senescent human CD8+ T cells. *PLoS One* 8:e64702. 10.1371/journal.pone.0064702 23717651 PMC3661524

[B11] DePianto D. J. Chandriani S. Abbas A. R. Jia G. N’diaye E. N. Caplazi P. (2014). Heterogeneous gene expression signatures correspond to distinct lung pathologies and biomarkers of disease severity in idiopathic pulmonary fibrosis. *Thorax* 70 48–56. 10.1136/thoraxjnl-2013-204596 25217476 PMC4472447

[B12] Dumeaux V. Olsen K. S. Nuel G. Paulssen R. H. Børresen-Dale A.-L. Lund E. (2010). Deciphering normal blood gene expression variation—The NOWAC postgenome study. *PLoS Genet.* 6:e1000873. 10.1371/journal.pgen.1000873 20300640 PMC2837385

[B13] Farsalinos K. E. Le Houezec J. (2015). Regulation in the face of uncertainty: the evidence on electronic nicotine delivery systems (e-cigarettes). *Risk Manag. Healthc. Policy* 8 157–167. 10.2147/RMHP.S62116 26457058 PMC4598199

[B14] Farsalinos K. E. Yannovits N. Sarri T. Voudris V. Poulas K. (2017). Nicotine delivery to the aerosol of a heat-not-burn tobacco product: comparison with a tobacco cigarette and e-cigarettes. *Nicotine Tob. Res.* 20 1004–1009. 10.1093/ntr/ntx138 28637344

[B15] Forster M. Liu C. Duke M. G. McAdam K. G. Proctor C. J. (2015). An experimental method to study emissions from heated tobacco between 100-200 degrees C. *Chem. Cent. J.* 9:20. 10.1186/s13065-015-0096-1 25941536 PMC4418098

[B16] Gautier L. Cope L. Bolstad B. M. Irizarry R. A. (2004). affy—analysis of Affymetrix GeneChip data at the probe level. *Bioinformatics* 20 307–315. 10.1093/bioinformatics/btg405 14960456

[B17] Gentleman R. C. Carey V. J. Bates D. M. Bolstad B. Dettling M. Dudoit S. (2004). Bioconductor: open software development for computational biology and bioinformatics. *Genome Biol.* 5:R80. 10.1186/gb-2004-5-10-r80 15461798 PMC545600

[B18] Hartung T. FitzGerald R. E. Jennings P. Mirams G. R. Peitsch M. C. Rostami-Hodjegan A. (2017). Systems toxicology: real world applications and opportunities. *Chem. Res. Toxicol.* 30 870–882. 10.1021/acs.chemrestox.7b00003 28362102 PMC5396025

[B19] Haziza C. de La Bourdonnaye G. Donelli A. Poux V. Skiada D. Weitkunat R. (2019). Reduction in exposure to selected harmful and potentially harmful constituents approaching those observed upon smoking abstinence in smokers switching to the menthol tobacco heating system 2.2 for three months (Part 1). *Nicotine Tob. Res.* 10.1093/ntr/ntz013 [Epub ahead of print]. 30722062 PMC7164581

[B20] Haziza C. de La Bourdonnaye G. Merlet S. Benzimra M. Ancerewicz J. Donelli A. (2016a). Assessment of the reduction in levels of exposure to harmful and potentially harmful constituents in Japanese subjects using a novel tobacco heating system compared with conventional cigarettes and smoking abstinence: a randomized controlled study in confinement. *Regul. Toxicol. Pharmacol.* 81 489–499. 10.1016/j.yrtph.2016.09.014 27693654

[B21] Haziza C. de La Bourdonnaye G. Skiada D. Ancerewicz J. Baker G. Picavet P. (2016b). Evaluation of the tobacco heating system 2.2. Part 8: 5-Day randomized reduced exposure clinical study in Poland. *Regul. Toxicol. Pharmacol.* 81 S139–S150. 10.1016/j.yrtph.2016.11.003 27816672

[B22] Hoonhorst S. J. Timens W. Koenderman L. Loi A. T. L. T. Lammers J.-W. J. Boezen H. M. (2014). Increased activation of blood neutrophils after cigarette smoking in young individuals susceptible to COPD. *Respir. Res.* 15:121. 10.1186/s12931-014-0121-2 25301367 PMC4203909

[B23] Huang H.-H. Liu X.-Y. Liang Y. Chai H. Xia L.-Y. (2015). Identification of 13 blood-based gene expression signatures to accurately distinguish tuberculosis from other pulmonary diseases and healthy controls. *Biomed. Mater. Eng.* 26 S1837–S1843. 10.3233/BME-151486 26405955

[B24] Jensen E. J. Pedersen B. Narvestadt E. Dahl R. (1998). Blood eosinophil and monocyte counts are related to smoking and lung function. *Respir. Med.* 92 63–69. 10.1016/S0954-6111(98)90034-8 9519227

[B25] Joseph P. Umbright C. Sellamuthu R. (2013). Blood transcriptomics: applications in toxicology. *J. Appl. Toxicol.* 33 1193–1202. 10.1002/jat.2861 23456664 PMC4550215

[B26] Kim C. H. Abedi M. Liu Y. Panuganti S. Flores F. Shah K. R. (2015). A novel technology for multiplex gene expression analysis directly from whole blood samples stabilized at ambient temperature using an RNA-stabilizing buffer. *J. Mol. Diagn.* 17 118–127. 10.1016/j.jmoldx.2014.11.002 25684272

[B27] Konstantopoulos S. Hedges L. V. (2009). “Analyzing effect sizes: Fixed-effects models,” in *The Handbook of Research Synthesis and Meta-Analysis*, eds Cooper H. Hedges L. V. Valentine J. C. (New York, NY: Russell Sage Foundation), 279–293.

[B28] LaBreche H. G. Meadows S. K. Nevins J. R. Chute J. P. (2011). Peripheral blood signatures of lead exposure. *PLoS One* 6:e23043. 10.1371/journal.pone.0023043 21829687 PMC3148235

[B29] Lucas J. Dressman H. K. Suchindran S. Nakamura M. Chao N. J. Himburg H. (2014). A translatable predictor of human radiation exposure. *PLoS One* 9:e107897. 10.1371/journal.pone.0107897 25255453 PMC4177872

[B30] Ludicke F. Picavet P. Baker G. Haziza C. Poux V. Lama N. (2018a). Effects of switching to the menthol tobacco heating system 2.2, smoking abstinence, or continued cigarette smoking on clinically relevant risk markers: a randomized, controlled, open-label, multicenter study in sequential confinement and ambulatory settings (Part 2). *Nicotine Tob. Res.* 20 173–182. 10.1093/ntr/ntx028 28177498 PMC5896432

[B31] Ludicke F. Picavet P. Baker G. Haziza C. Poux V. Lama N. (2018b). Effects of switching to the tobacco heating system 2.2 menthol, smoking abstinence, or continued cigarette smoking on biomarkers of exposure: a randomized, controlled, open-label, multicenter study in sequential confinement and ambulatory settings (Part 1). *Nicotine Tob. Res.* 20 161–172. 10.1093/ntr/ntw287 28177489 PMC5896533

[B32] Luke D. A. Ribisl K. M. Smith C. Sorg A. A. (2011). Family smoking prevention and tobacco control act. *Am. J. Prev. Med.* 40 295–302. 10.1016/j.amepre.2010.11.018 21335260

[B33] Martin F. Talikka M. Hoeng J. Peitsch M. (2015). Identification of gene expression signature for cigarette smoke exposure response—from man to mouse. *Hum. Exp. Toxicol.* 34 1200–1211. 10.1177/0960327115600364 26614807

[B34] Martin F. Talikka M. Ivanov N. V. Haziza C. Hoeng J. Peitsch M. C. (2016). Evaluation of the tobacco heating system 2.2. Part 9: application of systems pharmacology to identify exposure response markers in peripheral blood of smokers switching to THS2. 2. *Regul. Toxicol. Pharmacol.* 81 S151–S157. 10.1016/j.yrtph.2016.11.011 27845159

[B35] McCall M. N. Bolstad B. M. Irizarry R. A. (2010). Frozen robust multiarray analysis (fRMA). *Biostatistics* 11 242–253. 10.1093/biostatistics/kxp059 20097884 PMC2830579

[B36] McNeil A. (2012). “Reducing Harm from Nicotine Use,” in *Fifty Years Since Smoking and Health: Progress, Lessons and Priorities for a Smoke-free UK*, ed. Britton J. (London: Royal College of Physicians).

[B37] Murphy J. Liu C. McAdam K. Gaҫa M. Prasad K. Camacho O. (2017). Assessment of tobacco heating product THP1. 0. Part 9: the placement of a range of next-generation products on an emissions continuum relative to cigarettes via pre-clinical assessment studies. *Regul. Toxicol. Pharmacol.* 93 92–104. 10.1016/j.yrtph.2017.10.001 29080852

[B38] Poussin C. Belcastro V. Martin F. Boué S. Peitsch M. C. Hoeng J. (2017). Crowd-sourced verification of computational methods and data in systems toxicology: a case study with a heat-not-burn candidate modified risk tobacco product. *Chem. Res. Toxicol.* 30 934–945. 10.1021/acs.chemrestox.6b00345 28085253

[B39] R Development Core Team (2007). *R: A Language and Environment for Statistical Computing.* Vienna: R foundation for statistical computing.

[B40] Ringnér M. Staaf J. (2016). Consensus of gene expression phenotypes and prognostic risk predictors in primary lung adenocarcinoma. *Oncotarget* 7:52957. 10.18632/oncotarget.10641 27437773 PMC5288161

[B41] Rotunno M. Hu N. Su H. Wang C. Goldstein A. M. Bergen A. W. (2011). A gene expression signature from peripheral whole blood for stage I lung adenocarcinoma. *Cancer Prev. Res.* 4 1599–1608. 10.1158/1940-6207.CAPR-10-0170 21742797 PMC3188352

[B42] Schaller J.-P. Keller D. Poget L. Pratte P. Kaelin E. McHugh D. (2016a). Evaluation of the tobacco heating system 2.2. Part 2: chemical composition, genotoxicity, cytotoxicity, and physical properties of the aerosol. *Regul. Toxicol. Pharmacol.* 81 S27–S47. 10.1016/j.yrtph.2016.10.001 27720919

[B43] Schaller J.-P. Pijnenburg J. P. Ajithkumar A. Tricker A. R. (2016b). Evaluation of the tobacco heating system 2.2. Part 3: influence of the tobacco blend on the formation of harmful and potentially harmful constituents of the Tobacco Heating System 2.2 aerosol. *Regul. Toxicol. Pharmacol.* 81 S48–S58. 10.1016/j.yrtph.2016.10.016 27793747

[B44] Showe M. K. Vachani A. Kossenkov A. V. Yousef M. Nichols C. Nikonova E. V. (2009). Gene expression profiles in peripheral blood mononuclear cells can distinguish patients with non–small cell lung cancer from patients with nonmalignant lung disease. *Cancer Res.* 69 9202–9210. 10.1158/0008-5472.CAN-09-1378 19951989 PMC2798582

[B45] Smith M. R. Clark B. Lüdicke F. Schaller J.-P. Vanscheeuwijck P. Hoeng J. (2016). Evaluation of the tobacco heating system 2.2. Part 1: description of the system and the scientific assessment program. *Regul. Toxicol. Pharmacol.* 81 S17–S26. 10.1016/j.yrtph.2016.07.006 27450400

[B46] Smyth G. K. (2005). “Limma: linear models for microarray data,” in *Bioinformatics and Computational Biology Solutions Using R and Bioconductor*, eds Gentleman R. Carey V. J. Huber W. Irizarry R. A. Dudoit S. (New York, NY: Springer), 397–420. 10.1007/0-387-29362-0_23

[B47] Spira A. Beane J. Shah V. Liu G. Schembri F. Yang X. (2004). Effects of cigarette smoke on the human airway epithelial cell transcriptome. *Proc. Natl. Acad. Sci. U.S.A.* 101 10143–10148. 10.1073/pnas.0401422101 15210990 PMC454179

[B48] Spira A. Beane J. E. Shah V. Steiling K. Liu G. Schembri F. (2007). Airway epithelial gene expression in the diagnostic evaluation of smokers with suspect lung cancer. *Nat. Med.* 13 361–366. 10.1038/nm1556 17334370

[B49] Sturla S. J. Boobis A. R. FitzGerald R. E. Hoeng J. Kavlock R. J. Schirmer K. (2014). Systems toxicology: from basic research to risk assessment. *Chem. Res. Toxicol.* 27 314–329. 10.1021/tx400410s 24446777 PMC3964730

[B50] Tobacco Advisory Group of the Royal College of Physicians (2000). *Nicotine Addiction in Britain: a Report of the Tobacco Advisory Group of the Royal College of Physicians.* London: Royal College of Physicians.

[B51] Tobacco Advisory Group of the Royal College of Physicians (2016). *Nicotine Without Smoke—Tobacco Harm Reduction.* London: Royal College of Physicians.

[B52] U.S. Food and Drug Administration. (2012). Harmful and potentially harmful constituents in tobacco products and tobacco smoke; established list. *Fed. Regist.* 77 20034–20037.

[B53] U.S. Department of Health and Human Services (2014). *The Health Consequences of Smoking—50 years of Progress: a Report of the Surgeon General.* Atlanta, GA: US Department of Health and Human Services.

[B54] Viechtbauer W. (2005). Bias and efficiency of meta-analytic variance estimators in the random-effects model. *J. Educ. Behav. Stat.* 30 261–293. 10.3102/10769986030003261

[B55] Wang J. Urbanowicz R. A. Tighe P. J. Todd I. Corne J. M. Fairclough L. C. (2013). Differential activation of killer cells in the circulation and the lung: a study of current smoking status and chronic obstructive pulmonary disease (COPD). *PLoS One* 8:e58556. 10.1371/journal.pone.0058556 23505535 PMC3594304

[B56] Zander T. Hofmann A. Staratschek-Jox A. Classen S. Debey-Pascher S. Maisel D. (2011). Blood-based gene expression signatures in non–small cell lung cancer. *Clin. Cancer Res.* 17 3360–3367. 10.1158/1078-0432.CCR-10-0533 21558400

[B57] Zhu S.-H. Lee M. Zhuang Y.-L. Gamst A. Wolfson T. (2012). Interventions to increase smoking cessation at the population level: how much progress has been made in the last two decades? *Tob. Control* 21 110–118. 10.1136/tobaccocontrol-2011-050371 22345233 PMC3446870

